# Singular Cause of Death

**Published:** 1883-01

**Authors:** 


SINGULAR CAUSE OF DEATH.---Mr. William J. Thulman, a druggist of Buffalo, recently came to his death from a singular cause. While eating his dinner a large amalgam filling in one of his teeth became detached, and was swallowed. He immediately expressed his apprehension of trouble from it, but felt no special inconvenience for some days, when he began to experience pain in the abdominal region. The symptoms became aggravated, peritonitis ensued, and he finally died, after much suffering. An autopsy was held by prominent physicians, when it was found that the irregularly shaped mass had lodged in one of the lower folds of the ileum, and had produced an ulcer which had eaten its way through the intestines and finally caused his death.

